# Frequent Pet Contact as Risk Factor for Allergic Bronchopulmonary Aspergillosis in Cystic Fibrosis

**DOI:** 10.3389/fcimb.2020.601821

**Published:** 2021-01-11

**Authors:** Claudia Grehn, Patience Eschenhagen, Svenja Temming, Uta Düesberg, Konrad Neumann, Carsten Schwarz

**Affiliations:** ^1^ Department of Pediatric Pneumology, Immunology and Intensive Care Medicine, CF Center, Charité–Universitätsmedizin Berlin, Berlin, Germany; ^2^ Mukoviszidose Institut, Bonn, Germany; ^3^ Institute for Biometry and Clinical Epidemiology, Charité–Universitätsmedizin Berlin, Berlin, Germany

**Keywords:** aspergillosis, *Aspergillus fumigatus*, allergic bronchopulmonary aspergillosis, respiratory infection, pet, cystic fibrosis, cat, dog

## Abstract

*Aspergillus fumigatus* (*Af*) frequently colonizes the respiratory tract of patients with cystic fibrosis (CF). *Af* is associated with loss of pulmonary function and allergic bronchopulmonary aspergillosis (ABPA), a hypersensitivity fungal lung disease. Environmental factors have impact on CF patients’ lung function variation. The aim of this nationwide questionnaire survey was to investigate the amount of CF patients with frequent pet contact including pet species and to examine the potential impact of frequent pet contact on the occurrence of *Af* colonization and ABPA diagnosis in these patients. The survey was carried out in 31 German CF centers in 2018. A total of 1232 who completed the surveys were included, and statistical analysis was performed by chi-squared test. Within the study cohort 49.8% of subjects (n = 614; CF patients < 18years: 49.4%, n = 234; ≥ 18years: 50.1%, n = 380) reported frequent contact to pets, of which 60.7% reported frequent contact to dogs, 42.3% to cats and other animals. Of those with frequent pet contact, 71.8% (n = 441) had contact to one pet or more pets from the same family. *Af* colonization was not significantly associated with frequent pet contact. ABPA diagnosis was documented in 16.7% (n = 206) of all included CF patients and was significantly associated with frequent pet contact (18.9%, n = 116, p = 0.042), confirming previous single center examinations. Particularly, patients with frequent contact to dogs showed an increased ABPA prevalence of 21.3%. Frequent pet contact might be a risk factor for ABPA. CF patients who are sensitized to *Af* should be informed about the increased risk to develop an ABPA by frequent pet contact. Patients with recurrent onset of ABPA should be evaluated in terms of frequent pet contact.

## Introduction

Cystic fibrosis (CF) is a life-limiting recessive genetic disease. Mucus retention, chronic infections and inflammation in the airways lead to progressive respiratory impairment (Elborn, 2016). Beside bacterial species, fungal colonization is commonly observed in the respiratory tract of patients with CF (Ziesing et al., 2016). *Aspergillus fumigatus* (*Af*) is the most common filamentous fungus in CF (Ziesing et al., 2016; Schwarz et al., 2018). *Af* colonization is more common in adolescence and adulthood (Pihet et al., 2009; Warris et al., 2019). The respiratory tract of 10.3 to 60 % of CF patients is colonized by *Af* (Pihet et al., 2009; Ziesing et al., 2016; Warris et al., 2019; Hong et al., 2020). The presence of this environmental filamentous fungus in CF sputum is associated with worse respiratory quality of life (Hong et al., 2020). Inhaling *Af* spores into the lungs may cause multiples diseases including invasive pulmonary aspergillosis, aspergilloma (Mousavi et al., 2016) and growth of *Af* hyphae within the bronchial lumen triggers an immunoglobulin E (IgE)-mediated hypersensitivity response that results in airway inflammation, bronchospasm, and bronchiectasis (Armstead et al., 2014; Janahi et al., 2017). ABPA has a distinct Th-2 mediated pathophysiology and is associated with accelerated lung function decline (Tracy et al., 2016; Hong et al., 2020). ABPA is a frequent event in patients with CF (Tracy et al., 2016), with an age dependent occurrence (Maleki et al., 2020) and a prevalence of 3 to 25 % (Mastella et al., 2000; Patel et al., 2019; Maleki et al., 2020). Differences on reported rates of *Af* colonization and ABPA diagnosis might be influenced by regional variation in environmental load of *Af*, therapeutic regimes, seasonal or annual variation, the origin of samples (Ziesing et al., 2016). ABPA is challenging to diagnose and remains underdiagnosed in many patients (Janahi et al., 2017). ABPA is associated with increased lung function decline, more frequent hospitalizations and significant CF morbidity (Maturu and Agarwal, 2015; Keown et al., 2019). 45% of households in Germany have pets (Heimtierhaltung, 2018). Human contact with cats, dogs, and other pets results in several million pet-related infections each year. These parasitic, fungal, bacterial, viral or arthropod dependent infections range from self-limiting skin conditions to life-threatening systemic illnesses (Rabinowitz et al., 2007). Environmental factors have been shown to impact respiratory health. Exposure to environmental allergens like pet dander has been associated to worse respiratory outcome in other lung diseases such as asthma and pets may be potential sources for methicillin-resistant *Staphylococcus aureus* (MRSA) infection (Morrow et al., 2014). With regard to CF, case reports describe interspecies transmission of *Pseudomonas aeruginosa* (Mohan et al., 2008) and *Bordetella bronchiseptica* (Ner et al., 2003; Register et al., 2012) between pet cat/pet dog and CF patients. Furthermore, questionnaire data highlighted the association between cat ownership and higher frequency of nasal polyps as well as combined cat–dog ownership and higher rate of wheezing in CF patients (Morrow et al., 2014). The ubiquitous fungus *Af* possesses versatile features enabling them to survive in various environmental conditions, with a wide range of hosts including humans and animals (Mousavi et al., 2016). Retrospective single center data analysis reveals that ABPA is associated with pet ownership in CF (Thronicke et al., 2016). Therefore, pet ownership might pose a potential risk to patients with CF. From the perspective of preventive medicine, reservoirs of *Af* and the potential origin of infection in CF patients should be evaluated. Because of the difficulties of recognizing ABPA in the context of CF, due to overlapping clinical, radiographic, microbiologic and immunologic features (Stevens et al., 2003), advances in the understanding of possible risk factors may have a positive effect on patient prognosis. Limited work has been done examining fungal infections in CF regarding frequent pet contact. This questionnaire survey was conducted to determine frequent pet contact and pet species in CF, to examine the relationship between frequent pet contact and *Af* colonization as well as ABPA diagnosis in children and adult patients with CF.

## Materials and Methods

### Design and Development

This questionnaire survey was conducted at 31 German CF centers in 2018. The target population was patients with CF living in Germany. CF patients were recruited by their CF center team during their hospital stay independent of age and clinical status. Participation was voluntary. Ethical aspects were considered, and approval for the study was gained by the Ethics Committee of the Charité–Universitätsmedizin Berlin (EA2/057/18).

The questionnaire “Risk factors for *Af* infection in CF patients” included several items, which are not documented in the medical data base German Cystic Fibrosis Registry. Contact to pets (during last 12 months; several times per week, no/rare contact) and pet species were queried per questionnaire beside patient’s age (years) and sex (male, female, intersex), *Af* colonization within the last 12 months (negative, positive, multiple positive tests, unknown) and history of ABPA (negative, positive, unknown). For a positive of *Af* colonization within the 12 month of observation period, at least one positive microbiological indication was required. The ABPA diagnosis was determined in every single CF center and was based on the minimal 2003 Cystic Fibrosis Foundation (CFF) consensus criteria (Stevens et al., 2003). No other *Af* associated phenotypes were considered. Besides *Af*, no other *Aspergillus* spp. were included. Behavioral patterns, hygiene routines, onset, or history of pet contact and clinical data were not investigated. Only the part with respect to pet contact has been separately analysed for this manuscript. The questionnaire was sent to 94 CF treating centers in Germany in 2018 and data of 1,477 patients have been received from 31 CF centers.

### Statistical Analysis

Only completed questionnaires with distinct information on *Af* colonization, ABPA diagnosis and contact to pets were included in the final analysis. Frequent pet contact was defined as contact to animals/pets several times per week. Patients with no or rare animal/pet contact, *i.e.* less than once a week, were categorized as “no pet”. In the analysis, it was not distinguished whether the patients had contact to one animal or several animals of one species (*e.g.* fish). No scientific classifications were included for pets. In some cases, no further pet specifications were available, *e.g.* fish/aquarium, bird, reptile, or wild animal. Prevalence determination for single animal species was only performed for dogs and cats, due to case numbers. Means and standard deviations were calculated metrical variables. Frequency and percentage were used for categorical variables. Associations of categorical variables were analyzed with chi-square test. Since the study is exploratory no level of significance is specified, and the p-values are not Bonferroni adjusted. A p-value ≤0.05 is considered to be significant even if the first kind familywise error rate is not bounded by 5%. All figures were created using Graphpad Software, Inc. GraphPad Prism 8.4.0. All statistical analyses were performed using IBM SPSS Statistics Version 24 and Graphpad Software, Inc. GraphPad Prism 8.4.0.

## Results

A total of 1232 completed questionnaires were analyzed. Patients’ characteristics were shown in **Table 1**. 600 patients with CF (48.7%) were female. The mean age was 23 ± 14 years. Prevalence of 29.9% was found for *Af* and was significantly higher in adult patients (36.9%) compared to patients <18 years (18.6%; p < 0.001). Prevalence of 16.7% was found for ABPA. Likewise *Af* colonization, significantly higher values were observed in adults (22.7%) compared to children (7.2%; p < 0.001). N = 614 (49.8%) of the CF patients had frequent contact to pets. Among those, 234 patients were <18 years and 380 patients were ≥18 years of age. No difference was obtained in the frequency of frequent contact to pets in children (49.4%) and adults (50.1%; p = 0.794; **Table 1**).

**Table 1:** Patients characteristics.

	All CF patients	CF patients <18 years	CF patients ≥ 18 years	P value
	1232 (100.0%)	474 (38.5%)	758 (61.5%)	
Female sex, n (%)	600 (48.7%)	236 (49.8%)	364 (48.0%)	0.546
Age (years), mean ± SD (range)	23 ± 14 (0–88)	9 ± 5 (0–17)	31 ± 10 (18–88)	
*Af* colonization, n (%)	368 (29.9%)	88 (18.6%)	280 (36.9%)	<0.001
ABPA, n (%)	206 (16.7%)	34 (7.2%)	172 (22.7%)	<0.001
Frequent contact to pets, n (%)	614 (49.8%)	234 (49.4%)	380 (50.1%)	0.794

Of those with frequent contact to pets, 71.8% (n = 441) had contact to one pet (or more pets from the same family, *e.g.* fish; **Table 2**), 18.7% (n = 115) had contact to two pets and 7.0% (n = 43) had frequent contact to three up to five different pets. The animals mentioned in the questionnaire were listed in **Table 2**. The majority of CF patients had frequent contact to dogs (60.7%) and cats (42.3%), followed by horses and rabbits (**Table 2**).

**Table 2:** Pet contact in CF.

	Including all CF patients with frequent contact to pets, independent of the number of different pets (multiple choices possible) n = 614			Only CF patients included with frequent contact to a single pet or pets from one category (no multiple choices) n = 441		
	Number of CF patients, n (%)	*Af* colonization, n (%)	ABPA, n (%)	Number of CF patients, n (%)	*Af* colonization, n (%)	ABPA, n (%)
**Dog, cat**						
Dog	373 (60.7%)	118 (31.5%)	70 (18.7%)	240 (54.4%)	71 (29.6%)	51 (21.3%)
Cat	260 (42.3%)	92 (35.4%)	40 (15.4%)	152 (34.7%)	49 (32.2%)	25 (16.3%)
**Rabbit, rodent^a^**	68 (11.1%)	21	6	24 (5.4%)	9	2
**Horse, cattle, sheep, goat**	46 (7.5%)	18	8	9 (2.0%)	1	3
**Reptile, amphibian, spider^b^**	26 (4.2%)	11	9	6 (1.4%)	5	4
**Bird^c^**	18 (2.9%)	7	4	7 (1.6%)	0	1
**Fish^d^**	10 (1.6%)	2	1	2 (0.5%)	0	0
**Other^e^**	2 (0.3%)	1	1	0	0	0

^a^Rabbit, Hamster, Guinea pig, Gerbil, Mouse, Chinchilla. ^b^Tortoise, Frog, Reptile (no further specification), Chameleon, Iguana, Lizard, Newt, Snake, Spider. ^c^Budgerigar, Bird (no further specification), Chicken, Duck, Parrot. ^d^Fish, Aquarium (no further specification), Shrimp/Prawn. ^e^Hedgehog, Wild animal (no further specification).

**Figure 1:**
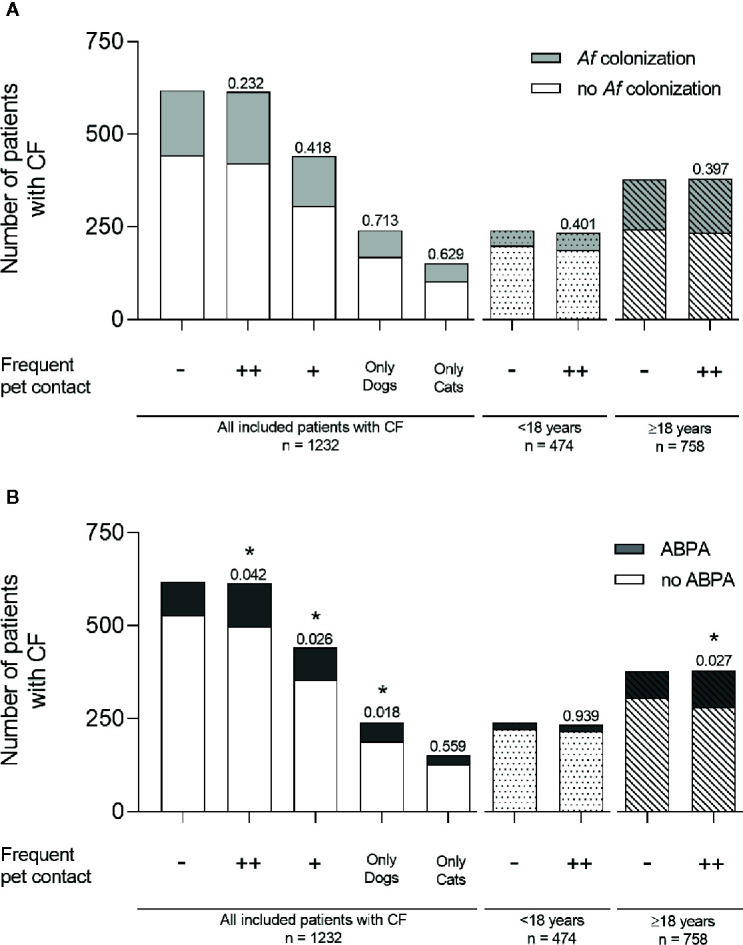
*Af* colonization and ABPA diagnosis in CF patients with or without frequent contact to pets. **(A)** *Af* colonization (light gray) and **(B)** ABPA diagnosis (dark gray) were shown for all included CF patients with no frequent pet contact (−), with frequent pet contact to one or more (++) different pets, with contact to only one pet (+), and with frequent contact to dogs only or cats only. Bars without fill pattern represent all included patients with CF. Dotted bars represent children aged under 18 years and striped bars represent adult patients ≥18 years. Significance levels of *p < 0.05 were compared to controls with no frequent pet contact. Statistical analysis was performed with chi-square test. Data are shown as absolute numbers.

The association of Af colonization with frequent pet contact missed significance (no pet n = 175; 28.3 %; Figure 1A). Af prevalence was similar for CF patients with frequent contact to pets (n = 193; 31.4 %; p = 0.232), with contact only to a single pet (n = 135; 30.6 %; p = 0.418), only dog pets (n = 71; 29.6 %; p = 0.713) or cat pets (n = 49; 32.2 %; p = 0.629; **Figure 1A**). In children a non-significant increase from 17.1 % (n = 41) to 20.1 % (n = 47) of Af colonization was observed in the context of frequent pet contact (p = 0.401; **Figure 1A**). In adults, Af prevalence increased from 35.4 % (no pet n = 134) to 38.2 % (pet contact n = 146), without reaching any significance (p = 0.397; **Figure 1A**).

ABPA was significantly more pronounced in patients with frequent pet contact (no pet n = 90; 14.6 %; pet contact n = 116; 18.9 %; p = 0.042; single pet n = 87; 19.7 %; p = 0.026; **Figure 1B**), in patients with frequent contact only to dogs (n = 51; 21.3 %; p = 0.018) and in adult patients with frequent pet contact (no pet n = 73; 19.3 %; pet contact n = 99; 26.1 %; p = 0.027; **Figure 1B**). No significant difference for ABPA diagnosis was observed in patients <18 years with frequent pet contact (no pet n = 17; 7.1 %; pet contact n = 17; 7.3 %; p = 0.939) and for patients with frequent contact only to cats (n = 25; 16.4 %; p = 0.559; **Figure 1B**). 

## Discussion

The present study explores frequent contact to pets, *Af* colonization and ABPA diagnosis in CF patients. 1232 patients with CF were included in the analysis. *Af* was documented in 368 and ABPA in 206 CF patients, both with significantly higher occurrence in adult individuals. Frequent pet contact was not found to be associated with *Af* colonization but was significantly associated with ABPA. Notably, the rate of frequent pet contact was similar in children and adult patients with CF. Our findings contribute to the limited literature on frequent pet contact and fungal infections in CF. A retrospective single centre analysis, including 55 pet owners, examined the association of ABPA with pet ownership (Thronicke et al., 2016). Due to the limited sample size, no correlation for ABPA (11 cases) in various pet groups were found (Thronicke et al., 2016). A wide range of animal species is kept as pets by CF patients, with dogs (60.7%) and cats (42.3%) as the most frequent ones (**Table 2**). This trend corresponds to the general tendency published elsewhere (Damborg et al., 2016). Environmental factors have been shown to impact respiratory health in CF (Morrow et al., 2014). After parasitic infections, fungal skin infections from cats and dogs are probably the most common pet-associated diseases (Rabinowitz et al., 2007). Case reports describe interspecies transmission of bacteria between cats/dogs and CF patients (Ner et al., 2003; Mohan et al., 2008; Register et al., 2012). Morrow et al. investigated cat and dog exposure in 703 CF patients (Morrow et al., 2014): 47.2% reported dog, and 28.1% reported cat ownership (Morrow et al., 2014). Combined cat–dog ownership was associated with wheezing, but no differences in lung function, self-reported environmental allergies, or ABPA were reported (Morrow et al., 2014). Here, a significant association between frequent contact to dogs and ABPA was observed (**Figure 1**). Due to missing information in the questionnaire, we cannot distinguish between different dog breeds or short/long haired dogs. Pet dogs have been considered to be involved in the contamination of indoor air by serving as a source of providing molds at houses (Jang et al., 2007). Both from skin and hairs, *Aspergillus* spp. was the most commonly found genus in dogs with isolation rates of 25% (Jang et al., 2007). Due to sample size limitations, other single pet groups were not included in statistical analysis (**Table 2**). In contrast to frequent pet contact, *Af* colonization and ABPA diagnosis were significantly pronounced in adult CF patients in this study (**Table 1**). In this nationwide, multicenter sample of CF patients, 49.8% reported to have frequent pet contact, mostly with one pet or pets from the same family (**Table 1**). This is slightly higher than documented in the general German population (45%) (Heimtierhaltung, 2018). Maybe, this circumstance is due to the fact, that frequent contact to pets has been associated with both emotional and physical health benefits (Carmack, 1991; Hemsworth and Pizer, 2006) especially in chronic illness and long-term conditions (Brooks et al., 2013). Furthermore, potential bias could result from particular interest of patients with frequent pet contact to participate in this study. Domesticated animals can affect the indoor microbiome by introducing exogenous microbial members into buildings (Leung and Lee, 2016). Pets may also act as vectors for various infectious agents (Rabinowitz et al., 2007). Close contact between pets and people offers favorable conditions for transmission by direct contact (*e.g.* petting, licking) or indirectly through contamination of domestic environments (Damborg et al., 2016). Even asymptomatic animals may transmit infections (Rabinowitz et al., 2007). To identify the reservoirs of *Af*, and thus a possible origin of infection in patients (Pihet et al., 2009) health care professionals should actively enquire about household pets and provide accurate information and practical advice on how to minimize the risk of infection (Hemsworth and Pizer, 2006; Rabinowitz et al., 2007). Although few studies have assessed the effectiveness of such measures, specific prevention guidelines involve common-sense measures, such as adequate handwashing and proper disposal of animal waste (Rabinowitz et al., 2007). Regarding high risk patients (*e.g.* immunocompromised patients including organ transplant recipients), contact with reptiles, including turtles, lizards, snakes as well as exotic and sick pets should be avoided (Rabinowitz et al., 2007). However, the benefits of the human-animal bond must be considered. Health care providers should be sensitive to the emotional attachment between patients and their pet and the psychological benefits of frequent pet contact (Carmack, 1991). With proper handling immunocompromised patients should be able to continue enjoying the significant benefits of frequent pet contact (Hemsworth and Pizer, 2006). In contrast to other studies (Morrow et al., 2014; Thronicke et al., 2016) the intensity of contact to pets was assessed and was defined as frequent contact, several times per week. This was implemented since the development of *Af* disease conditions is dependent on prolonged pathogen–host-interactions (Mousavi et al., 2016). Furthermore, a large sample size of 614 patients with frequent pet contact, including different age groups, was analyzed, and the number and species of pets present in the household was documented. The most significant limitation of our study is the absence of clinical data like lung function, serological markers, or allergy measures. Survey associated bias has to be considered. Moreover, the common behavioral patterns and hygiene routines associated with contact to pets were not enquired. Finally, the onset of pet exposure or history of pet contact was not collected. The potential influence of other environmental factors like different residential area types, will be discussed in detail in a separate article of this thematic special issue (see “*Urban life as risk factor for aspergillosis*”). Frequent contact to pets should be queried actively during clinical visits, and CF patients should be informed about the risk to develop an ABPA. Especially CF patients with recurrent onset of ABPA should be examined in terms of frequent contact to pets.

## Conclusion

Frequent pet contact might be a risk factor for ABPA in patients with CF. These results should be included in patient guidance and preventive measures, especially for *Af* sensitized patients or patients with recurrent ABPA.

## Data Availability Statement

The raw data supporting the conclusions of this article will be made available by the authors, without undue reservation.

## Ethics Statement

The studies involving human participants were reviewed and approved by the Ethics Committee of the Charité–Universitätsmedizin Berlin (EA2/057/18). Written informed consent to participate in this study was provided by the participants’ legal guardian/next of kin.

## Author Contributions

CG, PE, ST, UD, KN, and CS contributed to the conception and design of the study. UD and CG organized the database. KN and CG performed the statistical analysis. CG, PE, ST, UD, KN, and CS wrote the manuscript. All authors contributed to the article and approved the submitted version.

## Funding

This work was supported by the German Federal Ministry of Education and Research (BMBF) – Project InfectControl 2020 (“Art4Fun”, 03ZZ0813E).

## Conflict of Interest

The authors declare that the research was conducted in the absence of any commercial or financial relationships that could be construed as a potential conflict of interest.
